# Association of Awake Bruxism with Khat, Coffee, Tobacco, and Stress among Jazan University Students

**DOI:** 10.1155/2015/842096

**Published:** 2015-09-30

**Authors:** Mir Faeq Ali Quadri, Ali Mahnashi, Ayman Al Almutahhir, Hamzah Tubayqi, Abdullah Hakami, Mohamed Arishi, Abdulwahab Alamir

**Affiliations:** ^1^Department of Dental Public Health, College of Dentistry, Jazan University, P.O. Box 114, Jazan 45142, Saudi Arabia; ^2^College of Dentistry, Jazan University, P.O. Box 114, Jazan 45142, Saudi Arabia; ^3^Department of Diagnostic Sciences, College of Dentistry, Jazan University, P.O. Box 114, Jazan 45142, Saudi Arabia

## Abstract

*Objective.* The objective is to assess the prevalence of bruxism among the university students and to check its association with their khat chewing habit. *Materials and Methods.* A cross-sectional descriptive study is designed using cluster random sampling. Pretested questionnaire was administered by a trained interviewer to assess awake bruxism and the use of variables like khat, coffee, tobacco, and stress. Chi-square test at 5% significance was used for assessing the association. Logistic regression was also performed after adjusting for covariates. *Results.* A high response rate (95%) was obtained as the distribution and collection of questionnaire was within an hour interval. 85% (63%, males; 22%, females) experienced an episode of bruxism at least one time in the past six months. Regression analysis revealed an association of stress (*P* = 0.00; OR = 5.902, 95% CI 2.614–13.325) and khat use (*P* = 0.05; OR = 1.629, 95% CI 0.360–7.368) with bruxism. Interestingly, it is observed that the one who chew khat experienced 3.56 times (95% CI; 2.62–11.22) less pain when compared to the nonusers. *Conclusion.* This study is the first of its kind to assess the association of bruxism with khat chewing. High amount of stress and khat use can be considered as important risk indicators for awake bruxism.

## 1. Introduction

Bruxism, also known as tooth grinding, is a distinct type of oral habit possessing rhythmic activity of the orofacial muscles causing a vigorous contact between the surfaces of the teeth [1]. Although, there is no unanimous agreement on the definition of bruxism, some scholars defined it as a “repetitive jaw-muscle activity characterized by clenching or grinding of the teeth and/or by bracing or thrusting of the mandible” [2]. Bruxers are usually divided into the day time bruxers (awake bruxers) and the night time bruxers (sleep bruxers). Awake bruxism is a day time parafunctional activity of the muscles of mastication [3] and it is reported that usually the clenching of the teeth and grinding is loud enough for the partners to notice [1].

The dental health care practitioners are particularly concerned with bruxism because of the detrimental effect it has on the oral hard and soft tissues. Though there have been quite a few studies conducted on bruxism which portray its link with various factors, the confirmed cause of bruxism has not been determined till date [4]. In the early years, it was thought to occur due to occlusal interferences or problematic anatomy of a person; however, recent studies have shown specific factors to be linked to bruxism [4]. In one study conducted among the study sample of a developed nation, bruxism was reported to be associated with psychological stress, smoking, genetic factors, emotional conditions, and mental instability [5]. Some researchers have also shown its association with other parasomnias such as hallucinations and other violent injurious behaviors [6]. The modifiable risk factors reported to be closely linked to bruxism are coffee, tobacco, and alcohol [5]. All of these are known to have a high impact on the central nervous system of an individual.

Studies have reported a high use of some of these modifiable risk factors such as tobacco and coffee by people of all age groups in the Jazan region of Saudi Arabia [7]. Apart from these risk factors, the local population in this region of Saudi Arabia is also seen to be indulged in the habit of khat chewing on a regular basis [7]. Studies report that khat (*Catha edulis*) use is highly prevalent among the people living in the Eastern Africa and Southwestern Arabian Peninsula [7]. The fresh leaves of this plant are reported to have a similar effect on the central nervous system to the use of amphetamine [8]. Khat use may also lead to elevated blood pressure and increase in heart rate [9]. The world drug report in 2001 has presented Saudi Arabia among the top 5 countries which have the population of khat chewers [10], and in this country most of the khat chewers are reported to be from the Jazan region [7]. One study reported that the urban part of this region had more khat users when compared to the rural population [11]. The prevalence rate of khat users in the schools is seen to be 37.7% among the boys and 3.7% among the girls [12]. It is also reported that the khat use increases with age and number of years of study [13].

Thus, there is a lack of data on bruxism and its associated factors in the developing nations and at present there is no study which has evaluated the association of khat use with bruxism among any study population. The current study aims at assessing the prevalence of bruxism among the Jazan University students and checking its association with their khat chewing habit.

## 2. Materials and Methods

### 2.1. Ethical Considerations

The current study was approved by the ethics committee at Faculty of Dentistry, Jazan University, Saudi Arabia. An informed consent was taken from the participants before including them in the study.

### 2.2. Study Setting

This study was conducted in the Jazan University campus which is located at the southern tip of Saudi Arabia bordering Yemen. The participants were only Arab nationals making it a homogenized sample.

### 2.3. Study Design and Sampling

The current study is designed as a cross-sectional descriptive type using cluster random sampling technique without the intention of determining the cause and effect relationship. In the first stage of the study, the colleges under Jazan University were divided into various clusters and 4 colleges were selected randomly. The medical and dental wings were excluded as the students may have prior knowledge on bruxism which can lead to a bias. In the second stage, a simple random sampling of the participants among the selected colleges was performed. The participants who had no history of cervical or facial injury and who are not undergoing orthodontic treatment were included in the study.

### 2.4. Data Collection and Questionnaire

The questionnaire originally prepared in English was subjected to translation and reverse translation in the local language by bilingual dentists, who were fluent in both English and Arabic. Initially, a convenience sample size of 20 was randomly selected and the questionnaire was subjected to validity and reliability tests. To check the reliability of the questionnaire, a test/retest procedure and a measure of internal consistency using Cronbach-alpha coefficient were calculated [14] and it was found to be consistent as the minimum value of 0.68 was obtained. Apart from taking the sociodemographic details, the questionnaire was mainly divided into two sections. The first section consisted of questions pertaining to the list of signs and symptoms leading to the diagnosis of bruxism. It also had a direct question (“Have you ever been aware of clenching or grinding your teeth during wakefulness in the past 6 months?” yes/no) leading to the classification of the participants as a bruxer or a nonbruxer (awake bruxism). A similar type of questionnaire was previously used by Winocur and his colleagues [15]. The second part of the questionnaire consisted of the assessment of potential risk factors like coffee, khat, and tobacco use (both smoked and smokeless). These variables were dichotomized into “never users” or “ever users.” Stress was also assessed using an inventory which included symptoms like depression, strong heartbeat, dry mouth, angry burst, inability to concentrate, weakness, fatigability, muscle stiffness in the morning, lock jaws, tight jaws, pain in the jaws, insomnia, headache, and excessive sweating.

### 2.5. Statistical Analysis

All the data was stored and analyzed using SPSS version 20. The values were indicated as percentages or mean ± SD (standard deviation) wherever necessary. The chi-square test at 5% significance was used for assessing the association between variables. Logistic regression with 95th percentile was performed to determine the association between the variables.

## 3. Results

A total of 500 questionnaires were distributed and a high response rate of 95% (*N* = 476) was observed which can be attributed to the method of data collection. The session of questionnaire distribution and collection was on the same day during the break time of the respondents. Most of the male subjects fit the inclusion/exclusion criteria whereas few of their female counterparts had to be excluded as they had either prosthesis in their oral cavity or they were undergoing orthodontic treatment. So, the study sample had 71% males and 29% females. Most of the subjects (77%) had the age ranging from 21 to 25 years (Table 1). Subjective analysis revealed that 85% of university students (63% males and 22% females) had at least one episode of bruxism during the past six months. The university students between the ages of 21 and 25 were seen to be more affected with bruxism when compared to other age groups (Table 2). Also, the same age group (21–25 years) was seen to be more indulged in the use of khat, coffee, and tobacco when compared to the other age groups. Khat (36.9% to 14.9%), tobacco (31.7% to 16.6%), and coffee (60.1% to 24.8%) use was seen to be more in males when compared to females (Tables 1 and 2). The stress assessed in the study population was significantly on a higher side, with 87% of males and 89% of females reporting stress in their life (inclusive of relationships and academics).

About 47.2% of bruxers have reported pain in comparison with the nonbruxers (Table 4). In the analysis of multiple logistic regression, a strong association of stress (*P* = 0.00; OR = 5.902, 95% CI 2.614–13.325) and the khat use (*P* = 0.05; OR = 1.629, 95% CI 0.360–7.368) with bruxism was seen among the study sample (Table 3). Interestingly, it is observed that people who have the habit of chewing khat have nearly 3.56 times (95% CI; 2.62–11.22) less chance of developing pain when compared to the nonusers (Table 4). Thus, out of the variables assessed, only khat use and presence of stress were associated with the bruxism.

## 4. Discussion

Association of bruxism with khat among the university students was assessed for the first time in any study population. Bruxism is not a normal habit and in certain circumstances like when there is an increase in the frequency of episodes it may turn into a phenomenon with pathological consequences [16]. It is also reported that bruxism may dramatically alter results and the duration of the delicate and careful treatments performed by the dentists [16]. Hence, the current study is conducted in order to add to the literature on the field of bruxism. The study revealed that 85% of the university students had experienced at least one episode of bruxism during the past six months and this finding was high in comparison to another study conducted by Lavigne et al. [17]. The current study also portrays a strong association between bruxism and self-reported stress among the study population. Previous studies which investigated the relationship between bruxism and stress level had diverse results. Some studies in accordance with the current study confirmed the association [18–20] while other studies failed to confirm such correlation [6, 21]. In couple of other studies performed, the investigators showed that the high prevalence of bruxism among the police officers and the air force pilots was due to its strong association of the stress involved in their jobs [22, 23]. Thus, the high prevalence of bruxism among the Jazan University students can also be attributed to the high level of stress reported.

Khat (*Catha edulis*) use was assessed for the first time which showed a strong association with bruxism. There is no previous literature available till date to check such a relation, but there are some studies which were conducted to report the prevalence of khat among different age groups. One such study was done among secondary school children [7] and the prevalence results obtained were a little less when compared to its use among the university students examined in the current study. Khat use is considered as a public health problem in Saudi Arabia [24] and it is shown to affect the academic performance in children [24] and is also seen to be associated with psychosis in young adults [25, 26]. The significant relationship of khat use with bruxism suggests that more research work should be conducted, especially focusing on the cause and effect relationship.

A very interesting statistical finding in the current study was that the subjects who were in the habit of khat chewing were 3.56 times less likely of experiencing pain due to bruxism in comparison to the nonusers. This can be attributed to the cathinone content of the* Catha edulis* (khat), which has a similar composition and effect to amphetamine [27]. However, further investigations should be performed to confirm the association of khat in providing pain relief. Unlike other studies which demonstrated the association of tobacco and coffee use with bruxism [17], the current study population of university students showed no association. Out of the examined factors, only the stress attributed to the daily life and the khat chewing habit among the university students were seen to be significantly associated.

In the limitations of the study, it could be said that the assessment of bruxism through the questionnaire may not be accurate and has a chance of recall bias. The gold standard of evaluations [28] is expensive and may not have been feasible with the current budget of the study. The current method of assessment is widely acceptable by various researchers, both to diagnose and to check the association of potential risk factors with awake bruxism [16].

## 5. Conclusion

To conclude, it is observed that bruxism is highly prevalent among the Jazan University students. High amount of stress and khat use can be considered as important risk indicators for awake bruxism. Further studies are required in this field in order to portray the cause and effect association. Immediate public health measures should be taken to prevent the use of khat (*Catha edulis*) among the university students.

## Figures and Tables

**Table 1:** Distribution of the participants according to the variables assessed.

Variables	*N* (%)
Age	
Less than 15 years	2 (0.42%)
16–20 years	84 (17.64%)
21–25 years	366 (76.89%)
Above 25 years	24 (5.04%)
Gender	
Males	*N* = 339 (71.21%)
Females	*N* = 137 (28.78%)
Khat	
Users	*N* = 247 (51.89%)
Nonusers	
Tobacco	
Users	*N* = 230 (48.31%)
Nonusers	
Coffee	
Users	*N* = 404 (84.87%)
Nonusers	
Stress	
Yes	*N* = 422 (88.65%)
No	
Bruxism	
Bruxers	406 (85.29%)
Nonbruxers	70 (14.70%)

^*∗*^Percentages incorporated.

**Table 2:** Prevalence of bruxism and the use of potential risk factors among different age groups.

	Age groups				
	≤15	16–20	21–25	≥25	Total
Bruxers	2 (0.42%)	74 (15.54%)	312 (65.54%)	18 (3.78%)	406 (85.29%)
Khat	2 (0.42%)	33 (6.93%)	196 (41.17%)	16 (3.36%)	247 (51.89%)
Coffee	1 (0.21%)	71 (14.91%)	312 (65.54%)	20 (4.20%)	230 (48.31%)
Tobacco	0 (0%)	30 (6.30%)	183 (38.44%)	17 (3.57%)	230 (48.31%)
Stress	2 (0.42%)	74 (15.54%)	327 (68.69%)	19 (3.99%)	422 (88.65%)

**Table 3:** Association of risk factors with bruxism.

Risk factors (*N*)	Outcome				
	Bruxers	Nonbruxers	Mean ± SD	*P* value	OR^$^; (min-max)
Khat (247)	222 (46.6%)	25 (5.25%)	1.10 ± 0.30	0.05^*∗*^	1.629 (0.360–7.368)
Tobacco (230)	195 (40.96%)	35 (7.35%)	1.15 ± 0.35	0.67 (NS)	0.744 (0.249–2.221)
Coffee (404)	342 (71.84%)	62 (13.02%)	1.15 ± 0.36	0.40 (NS)	0.560 (0.253–1.237)
Stress (422)	405 (85.08%)	17 (3.57%)	1.04 ± 0.19	0.00^*∗*^	5.902 (2.614–13.325)

^*∗*^Significantly associated; NS: not significant; ^$^95% CI: confidence interval.

**Table 4:** Self-reported pain and its association with the use of coffee and khat.

	Pain	Yes	No	Mean ± SD	Odds ratio (OR)
Coffee users	Yes	225 (47.26%)	37 (7.77%)	1.14 ± 0.34	1.11 (NS)
	No	181 (38.02%)	33 (6.93%)	1.15 ± 0.36	

Khat users	Yes	100 (21%)	147 (30.88%)	1.59 ± 0.49	^*∗*^3.56 (95% CI; 2.62–11.22)
	No	162 (34.03%)	67 (14.07%)	1.29 ± 0.45	

^*∗*^Significantly associated; NS: not significant.
